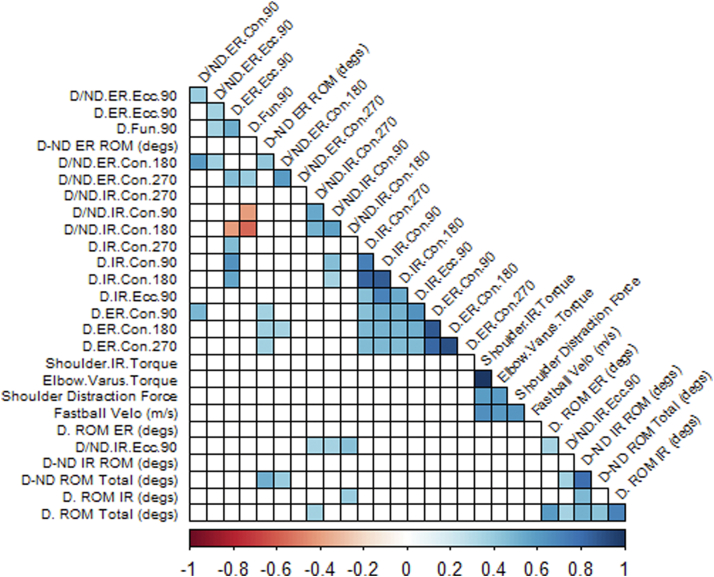# Clinical shoulder measurements related to joint loads in collegiate pitchers

**DOI:** 10.1016/j.xrrt.2022.09.004

**Published:** 2022-10-14

**Authors:** Aaron Trunt, David A. Sturdevant, Luke W. Adams, Nathan W. Skelley, Lisa N. MacFadden

**Affiliations:** a Sanford Sports Science Institute, Sanford Health, Sioux Falls, SD, USA; b Department of Biomedical Engineering, University of South Dakota, Sioux Falls, SD, USA; c Research Design and Biostatistics Core, Sanford Health, Sioux Falls, SD, USA; d Sanford Orthopaedics and Sports Medicine, Sanford Health, Sioux Falls, SD, USA

**Keywords:** Baseball, Range of motion, Isokinetics, Lasso regression, Rotator cuff, Torque

## Abstract

**Background:**

Pitchers are prone to upper extremity injury due to repetitive high joint loads. Clinical measures of shoulder strength and range of motion (ROM) have shown links to injury risk in pitchers, however, these factors have rarely been studied in relation to throwing joint loads. The purpose of this study was to identify which clinical ROM and isokinetic strength variables were related to peak shoulder and elbow joint torques in collegiate pitchers.

**Methods:**

Thirty-three healthy collegiate pitchers participated in this study. Fastball velocity, shoulder concentric and eccentric strength, and passive shoulder ROM variables were analyzed using a Lasso regression to determine what factors influenced shoulder internal rotation torque and elbow varus torque.

**Results:**

Fastball velocity was selected by the Lasso as indicator of increased shoulder and elbow torque. Passive shoulder external rotation ROM was also selected as an important factor in joint loading with increased shoulder external rotation ROM being related to lower joint loads. The bilateral ratio of shoulder internal rotator concentric strength was related to peak shoulder and elbow torques with an increase in the bilateral ratio of shoulder strength leading to reduced joint torques. Increases in the eccentric external rotator to concentric internal rotator strength (functional ratio) of the dominant arm and increases in dominant arm eccentric internal rotator strength were both related to increases in each joint torque.

**Conclusion:**

Results from the study indicate that pitch speed, passive shoulder external rotation ROM, and the isokinetic shoulder strength profile including internal rotator strength and functional strength ratio of pitchers are related to joint loading during the pitch and may be important to monitor in relation to injury risk and/or during rehabilitation. These results provide insight into the role that both shoulder ROM and rotator cuff strength play in the dynamic stabilization of the elbow and shoulder during pitching.

Injuries to the elbow and shoulder joints in baseball players are common across all ages.^10^^,^^20^^,^^39^ One contributing factor to upper extremity injuries in pitchers is that the baseball pitch is the fastest recorded human movement, capable of producing rotational speeds over 7000°/s.^39^ Due to the high speeds produced during the pitch and number of pitches a player typically throws, the shoulder and elbow undergo exceptionally high and repetitive loads during training and competition.^1^^,^^3^^,^^4^^,^^11^^,^^27^^,^^28^^,^^34^ Pitchers can generate joint torques greater than 100 N m and joint reaction forces of over 1000 N during every pitch.^12^ Because of these and other demands of the sport, musculoskeletal adaptations are commonly observed in the throwing arm of pitchers.

Beginning in adolescence, pitchers can exhibit adaptations in shoulder motion and strength.^19^^,^^21^ Pitchers often have increased external rotation and decreased internal rotation passively in the throwing shoulder compared to the non-throwing shoulder.^21^ Shoulder strength differences have also been well-documented with pitchers exhibiting increased internal rotator strength of the throwing shoulder without a corresponding increase in external rotator strength, lowering external-to-internal rotator strength ratios of the throwing arm compared to the non-throwing arm.^2^^,^^8^^,^^35^ While these adaptations are common in overhead athletes, for pitchers, such changes alter the biomechanics of the upper extremity and have been linked to injury risk and pain.^26^^,^^38^^,^^39^

Despite a wealth of literature on clinical measurements of upper extremity strength and motion in baseball pitchers, few studies have investigated the relationship between clinical shoulder characteristics and the biomechanical loads that the shoulder and elbow experience during the pitch. One study found that players presenting with glenohumeral internal rotation deficit (GIRD) compared to players without GIRD had no significant differences in elbow joint stress as measured by a wearable device.^30^ Other studies have found significant inverse relationships between throwing shoulder external rotation range of motion (ROM) and upper extremity joint moments in the pitch.^16^^,^^31^ Shoulder joint moments have also been found to be positively correlated to peak isometric strength of the internal rotators of the shoulder as measured by a handheld device.^16^ These previous studies have primarily used univariate analyses to determine relationships, when it is likely that these clinical factors related to joint loads are inter-related and may require more intricate analysis techniques. Furthermore, isometric measures of shoulder strength may be sufficient for determining the absolute strength of a given muscle group, but these methods do not take into account the dynamic nature of the throwing motion and associated demands placed on the musculature.

The term functional ratio has been defined previously as the strength ratio of the throwing shoulder’s eccentric external rotators and concentric internal rotators, which may be a more descriptive measurement of the dynamic strength a throwing athlete’s shoulder possesses.^23^^,^^35^ Specifically, the internal rotators act concentrically to accelerate the throwing arm and produce high ball speeds while the eccentric action of the external rotators are responsible for slowing down the arm after ball release and dissipating the large joint loads at the shoulder.^7^^,^^9^ Quantifying strength in this manner may lead to a deeper understanding of the role that dynamic shoulder strength plays in pitching. Isokinetic methods offer the ability to test shoulder strength both concentrically and eccentrically, but to our knowledge, only one study has examined the relationships between isokinetic shoulder strength and joint loading in pitchers.^5^ Therefore, the purpose of this study was to identify clinical variables, including ROM, isokinetics, and associated strength ratios, related to shoulder and elbow joint torques in the baseball pitch.

## Methods

Sanford Health’s Institutional Review Board approved this study (STUDY00001916). After providing informed consent, 33 collegiate baseball pitchers (age, 20.2 ± 1.4 y; height, 187.7 ± 6.5 cm; mass, 90.7 ± 12.4 kg) were recruited for this study. Four players were left-arm dominant, and the remaining 29 players were right-arm dominant. All participants were free of injury at the time of participation, including participants who had made a full return to baseball activities following past upper extremity injuries.

### Clinical measures

Basic anthropometrics (height and weight) were collected before each subject warmed up on an upper-body ergometer for 5 minutes at a self-selected pace. Subjects laid on a training table with the arm abducted to 90° where shoulder internal and external ROM were passively measured for both arms. Gamma et al found no significant differences in shoulder ROM following a traditional warmup in pitchers.^13^ Following established methods, ROM was collected with a standard long-armed goniometer with the scapular stabilization method to determine shoulder motion end range.^40^ One trained investigator performed all ROM testing procedures while another aligned the goniometer and recorded the measurements. Two trials were performed for each motion measured, and the order of testing for both arm and type of motion were randomized. If the two ROM measurements differed by greater than 10%, a third measurement was recorded, and the average of all recorded measurements was used for analysis.

**Figure 1:**
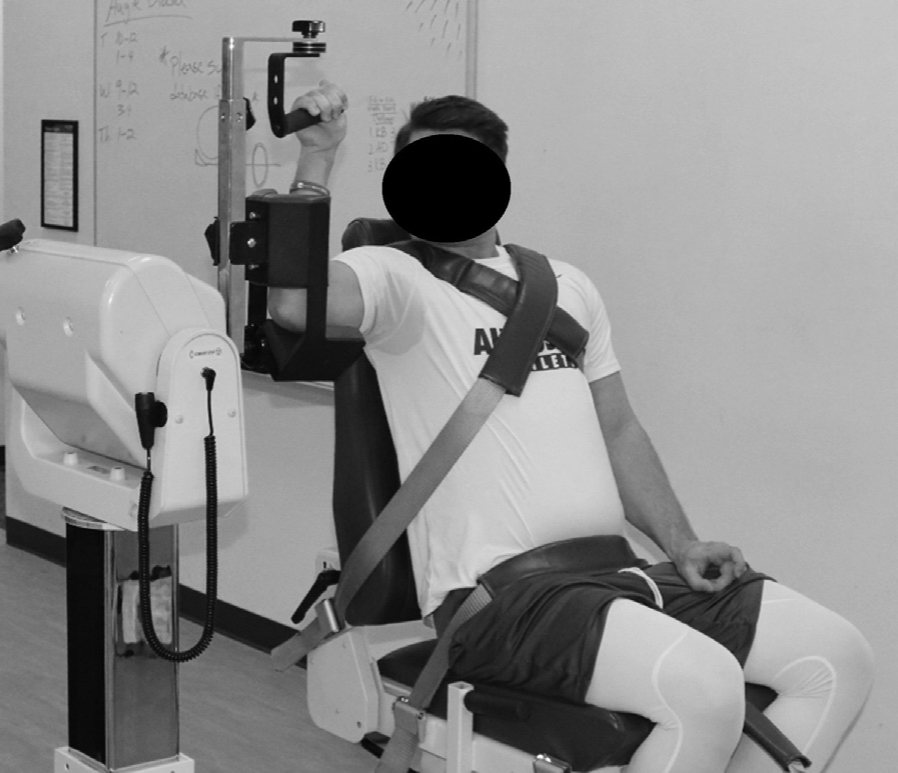
Example of isokinetic testing position. Testing arm was placed in the dynamometer at 90° shoulder abduction and 90° of elbow flexion in the scapular plane, as described previously.

**Figure 2:**
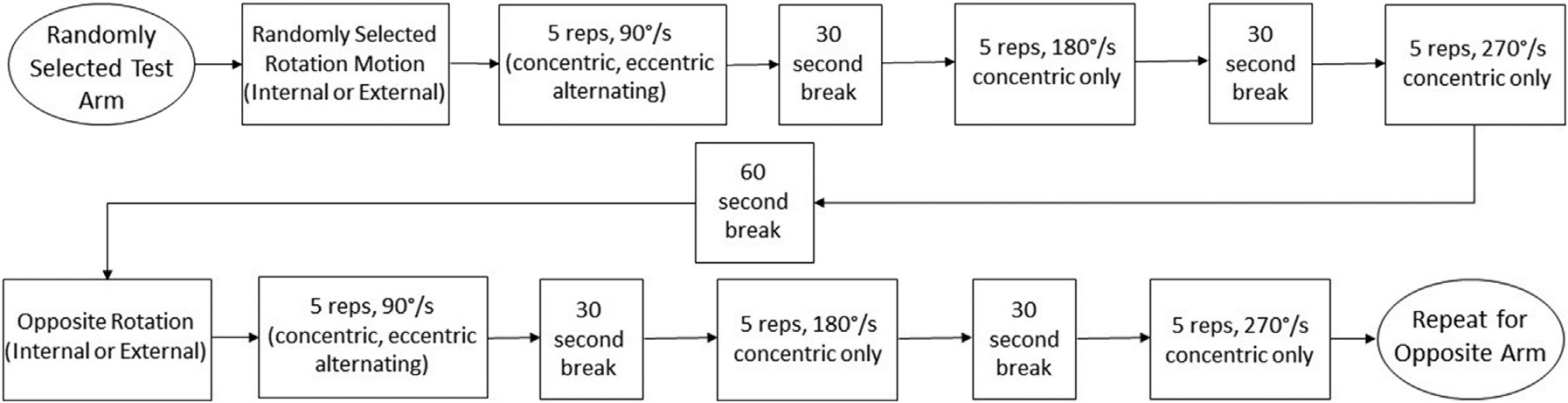
Isokinetic testing procedure for each subject. Initial test arm and muscle group were both chosen at random for each subject to avoid influence of fatigue/familiarity on the test results.

After completing ROM testing, the shoulder internal and external rotator strength for both arms was measured using an isokinetic dynamometer (Biodex System 4 Pro; Biodex Corp., Shirley, NY, USA). Participants were secured in the dynamometer with the arm positioned at 90° of shoulder abduction and 90° of elbow flexion in the scapular plane (Fig. 1). This position was selected over supine or neutral abduction because it provides a more accurate representation of the muscular strength and action necessary to compete in their given sport.^35^ Peak torque was assessed for concentric and eccentric action of the internal and external rotators for each arm at the 90°/s test speed and for concentric only at the 180°/s and 270°/s test speeds. The isokinetic testing procedure is described in Figure 2. Participants were given up to 5 practice repetitions at each speed to familiarize themselves with the protocol before completing 5 maximal-effort repetitions at the given speed. Peak torque normalized to bodyweight for each speed and muscle action for both arms was exported for analysis giving 16 individual peak torque measurements for each participant.

### Biomechanical data

Approximately 10 days after the clinical assessments, participants returned for data collection using motion capture at a biomechanics laboratory. No participants had been injured or participated in competition between testing dates and bullpen and training days were adjusted to accommodate the laboratory pitching session. All participants wore tight-fitting elastane shorts, indoor turf shoes, and their gloves. Participants completed a self-selected warmup typical of their pregame routine, which commonly consisted of dynamic and static stretching, throwing drills, and light tossing of baseballs. Next, participants threw warmup pitches until they indicated they were ready to pitch with full effort. All pitches were thrown from an artificial mound toward a net with a simulated strike zone as a target. Due to laboratory size constraints, the distance from the rubber to the strike zone was 13.7 m, shorter than regulation distance; however, the strike zone was scaled to account for the reduction in length.

**Figure 3:**
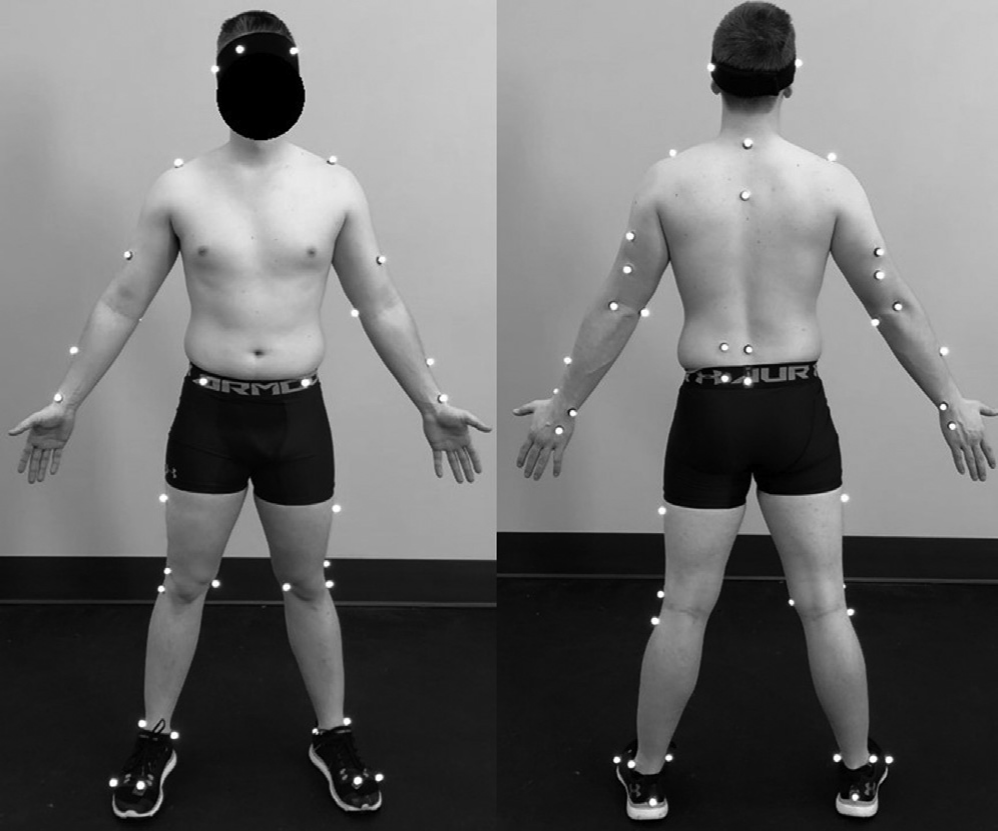
Example of the marker placement for all participants.

Following the warmup, a set of 50 retroreflective markers was applied to each subject using the Qualisys Baseball Performance marker set (Fig. 3). Reflective tape was placed on baseballs to identify ball release. Participants then threw a minimum of 10 full-effort fastballs while ball velocity, pitch location (strike or ball), and kinematic data were recorded. A radar gun (Stalker Pro II; Stalker Sports Radar, Plano, TX, USA) recorded pitch velocity from behind the strike zone. Three-dimensional motion data were collected using a 12-camera motion capture system (Oqus 7+; Qualisys Motion Capture Systems, Sweden) at 300 Hz. Raw marker trajectories were filtered using a fourth-order low-pass Butterworth filter with a cutoff frequency of 24 Hz. The 5 fastest pitches thrown for strikes were used for biomechanical analysis.

Kinematics and kinetics were calculated in Visual3D (C-Motion Inc., Germantown, MD, USA) using the motion data; estimated inertial properties of the arm, forearm, hand, and ball; and standard inverse dynamics equations.^6^^,^^11^^,^^18^ The baseball was modeled as a 0.142 kg point mass, fixed to the center of the hand, present until the instant of ball release. For each pitch, peak elbow varus torque and peak shoulder internal rotation (IR) torque were calculated. Kinetic variables were expressed internally, normalized to subject bodyweight and height for between-subject comparison, and averaged across the 5 pitches for analysis.^16^

### Statistical analysis

Shoulder and elbow kinetics were used as the two dependent variables, while subject ROM, isokinetics, and fastball velocity acted as the 28 independent variables. Due to the high-dimensional data and multicollinearity present in the clinical measurements (Supplementary Figure S1), a Lasso regression was chosen for analysis, as this method is ideal for handling data sets with these qualities.^25^^,^^32^^,^^33^ The Lasso works by minimizing the quadratic criterion penalized by the L_1_-norm of the coefficients. This use of penalization coupled with multiple replications reduces the variability in model estimation and effectively selects only independent variables most important for estimation of the given dependent variable. For each dependent variable, Lasso regression was performed using a 5-fold cross-validation method with 2000 bootstrap replications from the observed data's resampling and repeated over 100 different penalization parameter values to determine the model with the least mean squared error.^25^ Independent variables associated with the model having the minimum mean squared error were selected as the explanatory variables for each of the dependent variables. Coefficient estimates of the chosen independent variables for each of the 2000 replications were averaged, and an associated 95% confidence interval was calculated to determine model stability. Additionally, Pearson’s correlations were conducted to determine the linear relationships between each of the selected explanatory variables from the Lasso and their associated dependent variable.

## Results

Fastball velocity, two ROM measurements, and multiple isokinetic strength variables were selected by the Lasso indicating they are related to shoulder and elbow joint torque. Six variables were chosen from the best fit Lasso model for peak shoulder IR torque: fastball velocity, dominant arm passive external rotation ROM, the bilateral difference in arm external rotation ROM, dominant arm internal rotator peak torque measured eccentrically at 90°/s, the peak torque ratio between the dominant and nondominant internal rotators measured concentrically at 90°/s, and the dominant arm functional ratio measured at 90°/s. A complete list of all variables collected with definitions, means, and standard deviations can be found in Table I. Coefficients and 95% confidence intervals for each independent variable selected by the Lasso for the 2 models are given in Table II.

**Table I:** Means ± SD (standard deviation) and definitions of the 30 variables used for analysis.

Variable (units)	Mean ± SD	Definition
Peak shoulder internal rotation torque∗ (%)	7.10 ± 1.31	The peak joint moment at the shoulder during the cocking phase, expressed internally and normalized to body weight and height
Peak elbow varus torque∗ (%)	6.73 ± 1.17	The peak joint moment at the elbow during the cocking phase, expressed internally and normalized to body weight and height
**Fastball velocity (m/s)**	**37.53 ± 1.74**	Peak velocity of the baseball averaged across all five trials
Range of motion (°)		
**D.ER.ROM**	**126.23 ± 11.26**	Dominant arm external rotation range of motion
D.IR.ROM	47.68 ± 12.55	Dominant arm internal rotation range of motion
D.Total.ROM	173.88 ± 15.74	Sum of dominant arm internal and external range of motion
**D-ND.ER.ROM**	**9.62 ± 8.41**	Bilateral difference in shoulder external rotation range of motion
D-ND.IR.ROM	−14.44 ± 11.98	Bilateral difference in shoulder internal rotation range of motion
D-ND.Total.ROM	−4.51 ± 14.19	Bilateral difference in total shoulder range of motion
Dominant arm isokinetic peak torque (N·m/kg)		
D.ER.CON.90	0.46 ± 0.10	Concentric external rotators measured at 90°/s
D.ER.ECC.90	0.48 ± 0.15	Eccentric external rotators measured at 90°/s
D.IR.CON.90	0.73 ± 0.19	Concentric internal rotators measured at 90°/s
**D.IR.ECC.90**	**0.88 ± 0.22**	Eccentric internal rotators measured at 90°/s
D.ER.CON.180	0.39 ± 0.09	Concentric external rotators measured at 180°/s
D.IR.CON.180	0.63 ± 0.16	Concentric internal rotators measured at 180°/s
D.ER.CON.270	0.33 ± 0.10	Concentric external rotators measured at 270°/s
D.IR.CON.270	0.57 ± 0.19	Concentric internal rotators measured at 270°/s
Dominant/Nondominant arm isokinetic peak torque ratios (unit-less)		
D/ND.ER.CON.90	1.02 ± 0.16	Ratio of the concentric external rotators measured at 90°/s
D/ND.ER.ECC.90	1.21 ± 0.32	Ratio of the eccentric external rotators measured at 90°/s
**D/ND.IR.CON.90**	**1.12 ± 0.23**	Ratio of the concentric internal rotators measured at 90°/s
D/ND.IR.ECC.90	1.06 ± 0.16	Ratio of the eccentric internal rotators measured at 90°/s
D/ND.ER.CON.180	1.08 ± 0.23	Ratio of the concentric external rotators measured at 180°/s
**D/ND.IR.CON.180**	**1.15 ± 0.27**	Ratio of the concentric internal rotators measured at 180°/s
D/ND.ER.CON.270	1.22 ± 0.60	Ratio of the concentric external rotators measured at 270°/s
D/ND.IR.CON.270	1.24 ± 0.32	Ratio of the concentric internal rotators measured at 270°/s
Dominant arm agonist/antagonist isokinetic peak torque ratios (unit-less)		
D.ER/IR.CON.90	0.65 ± 0.18	Ratio of the concentric external to internal rotators measured at 90°/s
D.ER/IR.ECC.90	0.56 ± 0.20	Ratio of the eccentric external to internal rotators measured at 90°/s
D.ER/IR.CON.180	0.65 ± 0.18	Ratio of the concentric external to internal rotators measured at 180°/s
D.ER/IR.CON.270	0.62 ± 0.23	Ratio of the concentric external to internal rotators measured at 270°/s
**D.FUN.90**	**0.67 ± 0.17**	Functional ratio: the eccentric external rotators to concentric internal rotators measured at 90°/s

Bold indicates the variable was selected by the Lasso for one of the dependent variables.

Isokinetic and range of motion variables follow several rules: • The first letter(s) refer to the dominant arm, a ratio between arms, or a difference between arms. • Letters following the arm designation define type of ROM, muscle group tested, or a ratio of muscle groups. • The last three letters dictate muscle action(s) of the isokinetic test, or if ROM was assessed. • Ending numbers dictate isokinetic test speed, if present. • Example: D/ND.IR.CON.90 is the bilateral strength ratio of concentric internal rotator strength measured at 90º/s.

∗ Indicates dependent variables.

**Table II:** Variables selected by the Lasso for each dependent variable, sorted by ascending *P* value.

	FV (m/s)	D/ND.IR.CON.90	D-ND.ER.ROM (°)	D.FUN.90	D.ROM.ER (°)	D.IR.ECC.90 (N·m/kg)	D/ND.IR.CON.180
Mean	37.53	1.12	9.62	0.67	126.23	0.88	1.15
S.D.	1.73	0.23	8.41	0.17	11.26	0.22	0.27
Min	32.83	0.67	−11.5	0.32	102.00	0.56	0.64
Max	40.24	1.74	31.5	1.08	147.00	1.52	1.85
Peak shoulder IR torque (%)							
Lasso							
Coef.	0.343	−0.648	−0.012	0.374	−0.004	0.288	-
95% CI	[0.340, 0.346]	[−0.660, −0.636]	[−0.013, −0.012]	[0.355, 0.394]	[−0.004, −0.004]	[0.269, 0.308]	-
Pearson							
r	0.613	−0.321	−0.291	0.275	−0.264	0.229	-
*P*	<.001	.068	.099	.121	.138	.199	-
Peak elbow varus torque (%)							
Lasso							
Coef.	0.267	−0.440	−0.010	0.440	−0.003	0.339	−0.058
95% CI	[0.263, 0.270]	[−0.451, −0.430]	[−0.011, −0.010]	[0.420, 0.460]	[−0.004, −0.003]	[0.318, 0.360]	[−0.062, −0.055]
Pearson							
r	0.612	−0.312	−0.292	0.285	−0.255	0.229	−0.218
*P*	<.001	.078	.099	.102	.152	.200	.222

D.FUN.90, D/ND.IR.CON.90, and D/ND.IR.CON.180 are unit-less ratios.

*FV*, fastball velocity; *IR*, internal rotation; *CI*, confidence interval.

Seven variables were chosen from the best-fit Lasso model for peak elbow varus torque: fastball velocity, dominant arm passive external rotation ROM, the bilateral difference in arm external rotation ROM, dominant arm internal rotator peak torque measured eccentrically at 90°/s, the peak torque ratio between the dominant and nondominant internal rotators measured concentrically at 90°/s, the dominant arm functional ratio measured at 90°/s, and the peak torque ratio between the dominant and nondominant internal rotators measured concentrically at 180°/s. Results from the Pearson correlations revealed significant relationships between fastball velocity and the dependent variables with other independent variables selected by the Lasso approaching significance (Table II).

## Discussion

Multiple clinical factors in addition to fastball velocity were found to be related to peak elbow and shoulder torques. Fastball velocity showed the strongest correlation to both kinetic variables, suggesting pitch speed and joint loads of the throwing arm are closely related in collegiate pitchers. An inverse relationship existed between throwing shoulder external rotation ROM and both joint torques, demonstrating that pitchers with greater passive external rotation ROM of the throwing shoulder may experience lower joint loads during the pitch. Similar results were found for the bilateral difference in shoulder external rotation ROM, where a greater amount of external rotation ROM on the throwing arm compared to the non-throwing arm was linked to reduced joint loads. Additionally, collegiate pitchers exhibiting larger bilateral imbalances of shoulder internal rotator strength experienced less peak shoulder IR torque and less peak elbow varus torque during the pitch. Increased eccentric internal rotator strength of the throwing arm was linked to higher shoulder and elbow torques. Lastly, pitchers with a greater functional ratio in the throwing arm may also experience greater shoulder and elbow joint torques according to the results from the Lasso. These results may provide some insight into the role that dynamic muscle strength and ROM of the shoulder play in attenuating the upper extremity joint loads pitchers experience.

Pitch velocity has previously been linked to greater joint kinetics; however, the apparent influence of pitch speed on throwing arm kinetics varies with skill level.^15^^,^^22^^,^^24^^,^^29^ Fastball velocity was selected by the Lasso for both kinetic variables indicating that as pitch speed increases, so do the biomechanical loads in the throwing arm (Table II). In a study of high school-aged pitchers, there was a strong positive correlation between fastball velocity and peak elbow adduction moment.^15^ Post et al^24^ noted slightly weaker but significant correlations between fastball velocity and throwing arm kinetics while Nicholson et al^22^ noted similar correlations to the present study when looking at collegiate pitchers. In a retrospective study of professional pitchers, a significant but low correlation (*P* = .03, R^2^ = 0.076) between fastball velocity and maximum elbow varus torque was noted.^30^ These results suggest that pitch velocity accounts for some of the variance surrounding shoulder and elbow torques, but additional variance in throwing arm kinetics may be influenced by other factors, such as differences in throwing mechanics or clinical measures.

Passive ROM of the shoulder is a clinical measure that has been studied thoroughly in relation to injury risk and joint loading in pitchers.^16^^,^^17^^,^^30^^,^^36^^,^^37^ Although the presence of GIRD in the throwing arm has previously been considered a risk factor for injuries, more recent studies have identified insufficient external rotation of the throwing shoulder as a risk factor for throwing-related injury as well as a link to increased joint loading during the pitch.^31^^,^^36^ Increased external ROM on the throwing arm and a larger bilateral difference in external ROM decreased joint torques experienced in pitching according to the models. These findings are consistent with previous studies. Hurd and Kaufman^16^ noted significant inverse relationships between throwing shoulder external rotation ROM and peak shoulder and elbow joint moments in the pitch. Additionally, Wilk et al^36^ found that pitchers without a bilateral difference ≥5° in shoulder external rotation ROM favoring the throwing side were 2.2 times more likely to incur a shoulder injury resulting in lost playing time and 4 times more likely to undergo shoulder surgery. One reason for these findings could be that during the pitching motion the throwing shoulder undergoes active external rotation motion far greater than the passive motion measured clinically. Increasing passive shoulder external rotation motion may reduce the difference in active-passive external rotation thereby reducing the stress experienced at the shoulder. Given these prior studies and the current results, external rotation ROM should be monitored in the throwing arm and bilaterally in pitchers, as these measures may play a significant role in determining pitchers more susceptible to higher joint kinetics and increased injury risk.

Only one study has investigated isokinetic shoulder strength and joint kinetics in baseball pitchers, which identified isometric internal rotation strength and concentric external rotation strength as variables correlated with both peak shoulder compressive force and pitch velocity.^5^ The strength metrics from the prior study were similar to those found in our study and differed from values observed in the non-throwing athlete. These differences were consistent with those observed in previous studies such as disproportionate strength increases in internal vs. external rotators, in addition to increases in external rotation ROM and decreases in internal rotation ROM.^2^^,^^8^^,^^21^^,^^35^ The causes of these differences are multifactorial and include chronic adaptations to the repetitive forceful throwing motion such as humeral retrotorsion, glenoid retroversion, posterior rotator cuff and posterior capsular stiffness, among others.^14^ This previous study, however, only assessed strength and ROM in the throwing arm, which may neglect potentially useful clinical information such as bilateral strength ratios and ROM adaptations compared to the contralateral limb. An additional strength of the present study is the utilization of the Lasso regression technique for statistical analysis. The number of independent variables collected and presence of high multicollinearity within them (r > 0.8 for multiple pairwise comparisons) makes utilization of a penalization regression technique more appropriate than classical univariate tests. As more data are collected in biomechanical studies, it is likely that independent variables will outnumber the observations in a study and may be collinear. It is therefore necessary to employ statistical methods which can reduce the dimensionality of the data without overfitting the model. The Lasso regression offers this flexible solution by reducing the independent variables to a subset that is most important for estimation of the dependent variable, and as such, is an intriguing method to pursue in similar studies.

While some studies have found links between shoulder strength and joint torques in pitchers as measured with a handheld dynamometer, this is one of the first studies to identify these relationships using isokinetic dynamometry, which provides accurate measurements of both eccentric and concentric muscle actions. Pitchers with increased eccentric internal rotator strength in the throwing shoulder experienced higher shoulder and elbow joint torques during the pitch. The internal rotators of the throwing arm are acting eccentrically during the transition from the arm cocking to the arm acceleration phase of the pitch.^9^ Therefore, pitchers with stronger eccentric internal rotator muscles are likely able to increase the rotational acceleration of the throwing arm internally during this transition period, which may lead to greater shoulder and elbow joint torques experienced. A greater functional ratio was associated with increased joint torques in both the shoulder and elbow. A greater functional ratio could be due to higher eccentric external rotator strength and/or lower concentric internal rotator strength in the throwing arm. In our data set, eccentric external rotator strength showed a stronger correlation to the functional ratio, so it is likely a greater functional ratio is due to increased eccentric external rotator strength of the throwing arm rather than decreased concentric internal rotator strength (Supplementary Figure S1). Given the role of the external rotators in maintaining the humerus’s externally rotated position in the late cocking phase, it is possible that pitchers with stronger external rotators are able to keep the throwing arm’s externally rotated position longer into the throwing motion potentially increasing the stress on the elbow and shoulder as the acceleration phase begins. Still, the role of the eccentric/concentric rotator cuff strength in pitching biomechanics and injury mechanisms requires further study as early identification of these modifiable qualities in pitchers is key to injury risk reduction.

This study presents a novel link between bilateral isokinetic shoulder strength in collegiate pitchers and joint kinetics. Pitchers with increased dominant arm internal rotator strength compared to the nondominant arm experienced less elbow and shoulder torque than those without this muscular imbalance. The pitchers in this study exhibited concentric internal rotator strength in the dominant arm 12%-24% greater than that of the nondominant arm on average, depending on isokinetic test speed. Therefore, it is recommended that pitchers with concentric internal rotator strength of the throwing arm less than that of the contralateral arm work to strengthen the internal rotator musculature as these adaptations may be useful for attenuating joint kinetics in the pitch. These findings may also provide important rotator cuff strength guidelines for throwing athletes rehabbing an upper extremity injury. Clinicians should monitor bilateral internal rotator strength of the shoulder during the preseason and playing season as a method of estimating the stress that the athlete’s shoulder and elbow may be undergoing during throwing.

This study is not without limitations. The sample size consisted of 33 collegiate pitchers. Future studies should explore the role of isokinetic strength in relation to joint loads and injury risk in other populations of pitchers such as youth, high school, and professional. Pitchers also threw in a laboratory setting from a reduced distance compared to regulation. While laboratory settings differ from in-game scenarios, it is not feasible to use the current gold standard marker-based motion capture in a game. Additionally, a reduction in mound distance may limit the generalizability of these findings. Although no participants engaged in competition between assessments, and training and throwing sessions were scheduled to accommodate data collection, it is possible the time between assessments could influence biomechanics testing results. Lastly, even though many independent variables were collected for the study, the Lasso method only chose variables that were most influential to the dependent variable, reducing the likelihood of overfitting the model. Still, these model estimates should be interpreted with caution as there are likely many factors not included in this study that may influence pitching biomechanical loads.

## Conclusion

This study identified important clinical factors related to throwing biomechanical loads in a group of collegiate pitchers. Fastball velocity, shoulder external rotation ROM, and various isokinetic strength characteristics of the shoulder musculature were linked to increased shoulder and elbow joint torques in collegiate pitchers. Specifically, eccentric internal rotator strength of the throwing arm, the bilateral ratio of concentric internal rotator strength, and the functional strength ratio of the throwing arm were related to shoulder and elbow joint torques. It is recommended that clinicians monitor passive shoulder external rotation ROM, bilateral internal rotator strength, functional ratios, and sudden changes in pitch speed as these may increase biomechanical loading of the shoulder and elbow during pitching.

## Acknowledgments

The authors would like to thank Kyle Johnson, Jason Thompson, Zadok Isaacs, and Cody Reed for their help in the data collection processes, and Travis Burgers for his help in the review process.

## Disclaimers:

Funding: This study was internally funded by Sanford Health.

Conflicts of interest: The authors, their immediate families, and any research foundation with which they are affiliated have not received any financial payments or other benefits from any commercial entity related to the subject of this article.

## Figures and Tables

**Supplementary Figure S1:**